# Case Report: Congenital Perineal Lipoma Associated With Additional External Genitalia Anomalies

**DOI:** 10.3389/fped.2022.923801

**Published:** 2022-06-30

**Authors:** Francesca Tocchioni, Chiara Caporalini, Annamaria Buccoliero, Flavio Facchini, Marco Ghionzoli, Francesco Morini

**Affiliations:** ^1^Department of Pediatric and Neonatal Surgery, Meyer Children’s Hospital, Florence, Italy; ^2^Pathology Unit, Meyer Children’s Hospital, University of Florence, Florence, Italy

**Keywords:** lipoma, perineal mass, labioscrotal fold, accessory scrotum, children

## Abstract

Perineal lipoma is an uncommon congenital benign tumor sometimes associated with genitourinary or anorectal malformations. Accessory scrotum and accessory labioscrotal fold are infrequent features, often concurrent with perineal tumors. We describe a single institution experience with three consecutive cases of perineal lipoma associated with external genital anomalies, and a literature review.

## Introduction

Congenital lipoma is a benign tumor, which rarely occurs in infants, usually involving the trunk, nape, abdomen, forearms, buttocks, and thighs, while it is infrequent on the face, scalp and calves (1). A perineal position is sporadic and is generally associated with other genitourinary or anorectal anomalies (2–6), whereas isolated forms are anecdotal (1).

Congenital malformations of the external genitalia are uncommon anomalies, classified into four categories: penoscrotal transposition, bifid scrotum, ectopic scrotum and accessory scrotum in males or accessory labioscrotal fold in females (2). Among these, accessory scrotum/labioscrotal fold is the rarest anomaly (3) and may occur as an isolated malformation (7) or associated with other abnormalities such as lipoma, lipoblastoma, and hamartoma (2–4, 8). Herein, we present a single institution experience of three cases of perineal lipoma associated with external genitalia abnormalities. In addition, we performed a thorough literature search on the PubMed electronic database for English language reports using the following MeSH terms: “perineal lipoma,” “accessory scrotum” AND “perineal lipoma with accessory scrotum”/“labioscrotal fold.” From 1990 to 2021, sixteen reports on perineal lipoma associated with accessory scrotum or accessory labioscrotal fold were identified and reported (Table 1).

**TABLE 1:** Characteristics of the patients with perineal lipoma associated with accessory scrotum or accessory labioscrotal fold reported in the literature from 1990 to 2022 including our three cases.

References	Gender	Age	Mass location	Histology	Associated anomalies
Shimotake (21)	M	2 year	Left asymmetrical midline of perineum	Lipoma with AS	
Sule et al. (16)	F	1 month	Midline of perineum	Lipoma with ALF	
Sule et al. (16)	M	3 week	Midline of perineum	Lipoma with AS	
Redman et al. (13)	F	At birth	Posterior part of the left labium majus.	Adipose tissue	
Goktas (22)	M	40 year	Left asymmetrical midline of perineum	Lipoma with AS	
Park and Hong (2)	M	7 month	Midline of perineum	Lipoma	PST, bifid scrotum
Park and Hong (2)	M	4 month	Left asymmetrical midline of perineum	Lipoma	Asymmetrical PST
Harada (23)	M	4 year	Midline of perineum	Lipoma with AS	Meningocele
Soccorso (24)	M	1 year	Midline of perineum	Lipoma with AS	Pseudo-diphallus
Numajiri et al. (11)	F	4 year	On the right labium majus	Lipoma with ALF	
Numajiri et al. (11)	F	7 year	On the right labium majus	Lipoma with ALF	
Numajiri et al. (11)	F	3 year	Posterior part of the left labium majus	Lipoma with ALF	
Chatterjee (25)	M	1 year	Right asymmetrical midline of perineum	Lipoma with AS	
Kavecan (26)	M	1 month	Midline of perineum	Lipoma with AS	
Iida et al. (19)	M	18 month	Posterior part of the right scrotum	Lipoma with AS	Buried penis
Mifsud (15)	F	18 month	Left labial mass, which extended posteriorly	Lipoma	Accessory phallus. Absent right kidney, ectopic ovaries
Murase et al. (3)	M	1 month	Midline of perineum	Lipoma and AS	
Hashizume et al. (14)	F	At birth	Right labium majus	Adipose tissue	Anovestibular fistula
Fathaddin (27)	M	6 month	Above the anal orifice	Lipoma with AS	CPAM
Wang et al. (10)	6F, 1M	5–12 months	Lateral and mid-perineum	Lipoma with AS/ALF	Anorectal malformation, hypospadias with caudal duplication syndrome.
Tocchioni (28)	M	2 week	Perineal mass under the right hemiscrotum	Lipoma with AS	Incomplete PST, right penoscrotal fusion
Tocchioni (28)	F	6 month	Mass arising from the right labium major and perineum	Lipoma with ALF	Congenital hip dysplasia
Tocchioni (28)	F	2 year	Mass arising from the left labium major	Lipoma with ALF	

*AS, accessory scrotum; ALF, accessory labioscrotal fold; PST, penoscotal transposition; CPAM, congenital pulmonary airways malformation.*

## Case Report

### Case 1

**FIGURE 1:**
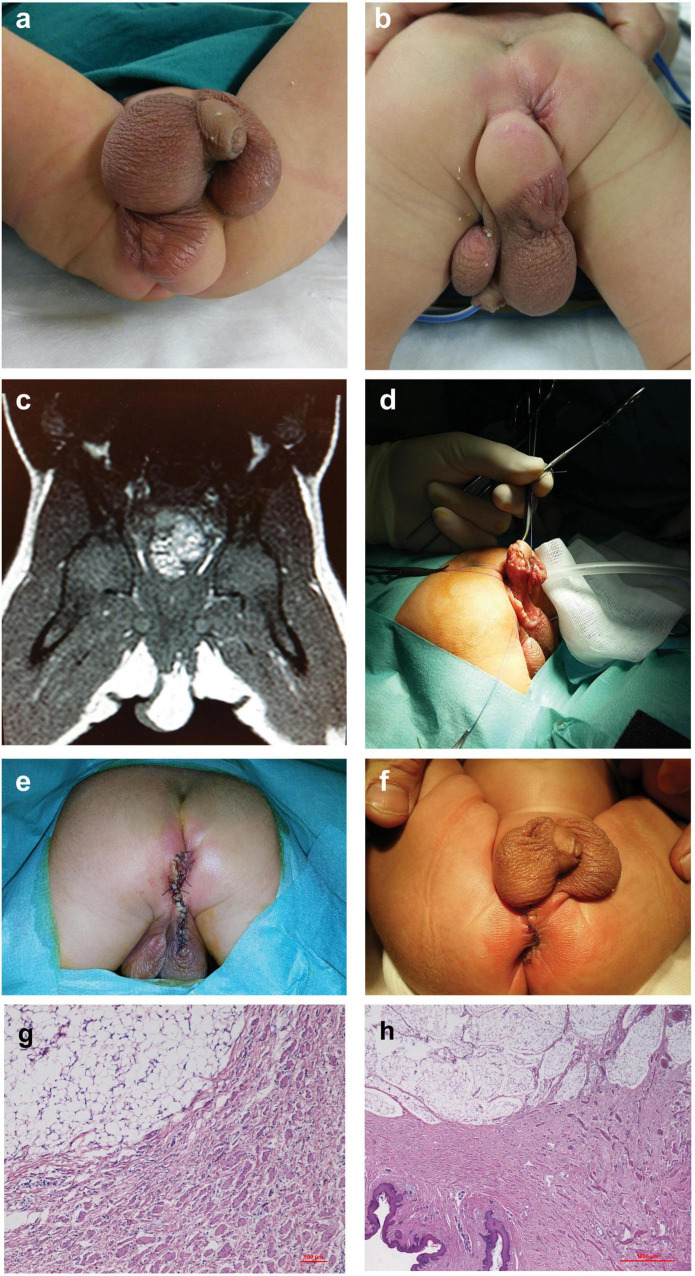
Case 1. **(a,b)** Soft and spherical mass under the right hemiscrotum, with rugated pigmented scrotal skin on its upper part associated with incomplete penoscrotal transposition and right penoscrotal fusion. **(c)** Abdominal MRI: exophytic adipose tissue mass. **(d)** Surgery: excision of the mass and **(e)** perineal closure through interrupted resorbable stitches. **(f)** One-month follow-up. **(g,h)** Histological examination of specimens showing an area characterized by smooth muscle bundles dispersed in dermal collagen and a contiguous area with an abundant mature adipose tissue in the deep dermis and hypodermis.

A full-term male neonate was referred to our service because of a soft, spherical, perineal mass under the right hemiscrotum, about 3 cm in diameter, and with a rugated and pigmented skin area on its upper part continuing to the scrotal skin. He also presented with incomplete penoscrotal transposition, right penoscrotal fusion, and right hydrocele (Figures 1a,b). The baby underwent abdominal ultrasonography and barium enema to rule out colorectal abnormalities, genetic and endocrinology evaluations, and blood test, which were normal. MRI showed an exophytic adipose tissue mass (Figure 1c). At 2 weeks of age, he underwent surgical excision (Figures 1d,e). On histolopathological analysis, the resected lesion proved to be cutaneous and subcutaneous tissues showing areas with different histological features: a smooth muscle bundles dispersed in the dermal collagen and an abundant mature adipose tissue in the deep dermis and hypodermis (Figures 1g,h), which were compatible with the diagnosis of accessory scrotum with lipoma. The post-operative course was uneventful, and the patient was discharged home after 6 days. The cosmetic outcome at 1-month follow-up was deemed good (Figure 1f). For this patient, penoscrotal transposition and fusion correction were scheduled after 1 year of age.

### Case 2

**FIGURE 2:**
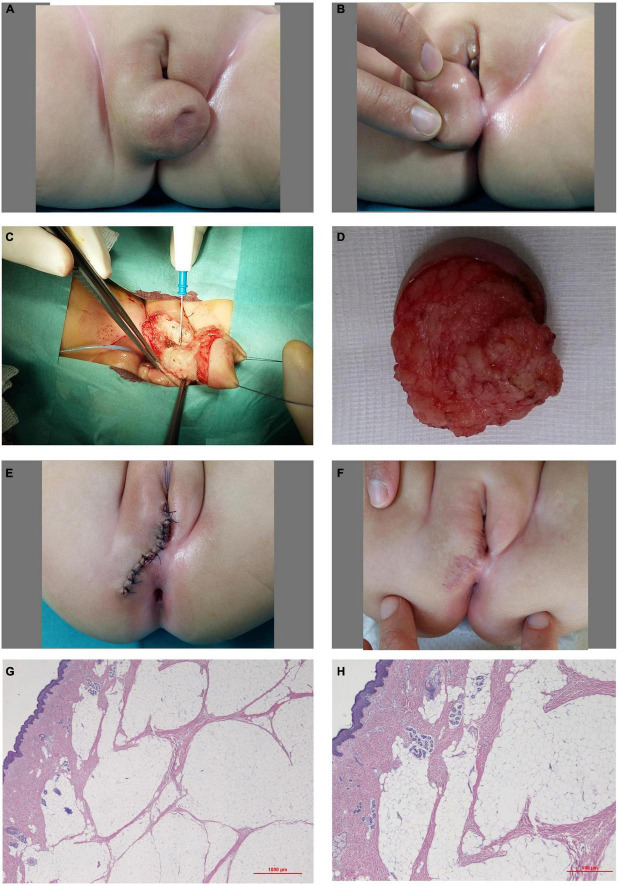
Case 2. **(A,B)** Soft and spherical mass arising from the caudal aspect of the right labium major and from the perineum with a hollow and wrinkled skin area on its center side. **(C)** Surgery: excision of the mass. **(D)** Surgical specimen. **(E)** Perineal and labium skin closure through interrupted resorbable stitches. **(F)** One-month follow-up. **(G,H)** Histological examination: abundant mature adipose fat cells and superficially intra-lesional entrapped adnexal structures.

A full-term female newborn was referred to our center for a perineal mass and congenital hip dysplasia. Physical examination showed a soft, spherical mass arising from the caudal aspect of the right labium major and from the perineum, measuring about 3 cm in diameter with a wrinkled skin area on its central side (Figures 2A,B). Abdominal ultrasonography, genetic and endocrinology evaluations and blood tests were normal. She underwent complete excision of the mass at 6 months of age (Figures 2C–E). The resected lesion consisted of cutaneous and subcutaneous tissues with abundant mature adipose cells and smooth muscle bundles dispersed in the dermal collagen and superficial adnexal structures (Figures 2G,H). The histological diagnosis was lipoma with accessory labioscrotal fold. The post-operative course was uneventful, and the patient was discharged home after 4 days. The cosmetic outcome at 1-month follow-up was excellent (Figure 2F).

### Case 3

**FIGURE 3:**
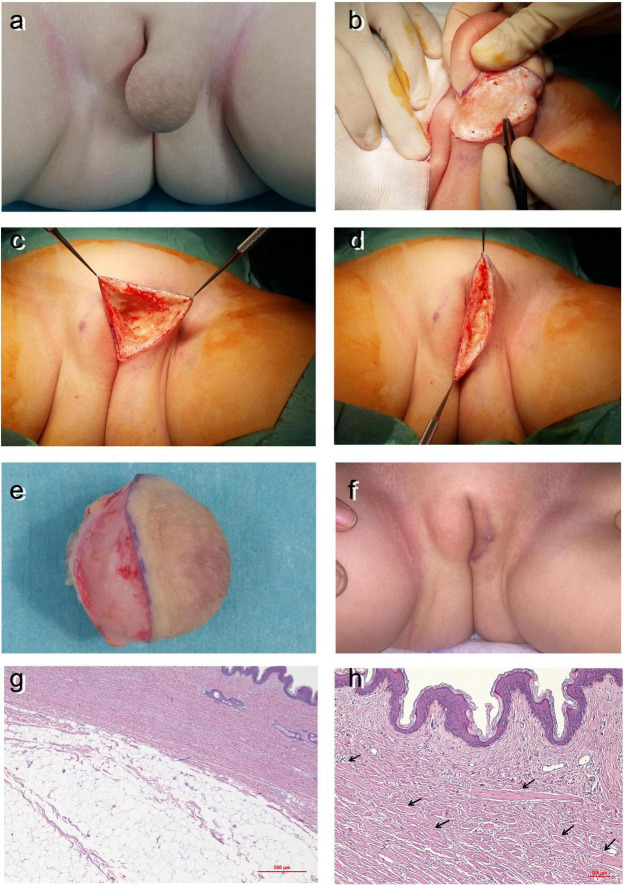
Case 3. **(a)** Soft and spherical mass arising from the central and caudal aspects of the left labium major with wrinkled unpigmented skin above. Surgery: **(b)** excision of the mass and **(c)** the labium major after mass excision. **(d)** Suture edges conform to continuous resorbable stitches. **(e)** Surgical specimen. **(f)** One-month follow-up. **(g,h)** Histological examination: abundant mature adipose fat cells both in the deep dermis and hypodermis and smooth muscle bundles in the dermis (arrows).

A 2-year-old girl was referred as an outpatient for a congenital soft, spherical mass arising from the central and caudal aspects of the left labium major. The mass measured about 5 cm in diameter and was covered by wrinkled, unpigmented, and scrotum-like skin: (Figure 3a). Diagnostic investigations were normal. The baby underwent surgical resection of the mass (Figures 3b–e). The excised lesions consisted of cutaneous and subcutaneous tissues characterized by the presence of abundant mature adipose fat cells both in the dermis and the hypodermis with smooth muscle bundles in the dermis, as shown by the arrows in Figures 3g,h. Also, in this patient, the histopathological diagnosis was lipoma with accessory labioscrotal fold. The post-operative course was uneventful, and the patient was discharged home after 1 day. The cosmetic outcome at 1-month follow-up was excellent (Figure 3f).

Informed consent to report and publish patient information and images was provided by the parents of all the patients.

## Discussion

We report our experience with perineal lipomas associated with external genitalia malformations and a review of the literature on these anomalies.

Congenital masses arising from the perineum, previously described under a variety of different names, such as lipoma, hamartoma, lipoblastoma, choristoma, sacrococcygeal teratoma, perineal hernia, and vascular anomalies, are uncommon but usually benign (2–4, 6, 8). In our series, none of the specimens demonstrate lobular architecture with adipocytes sheets and fibrovascular areas mingled with mixoid ones, typical of lipoblastoma. Additionally, we did not find an abundant fibrous tissue, which is a distinctive feature of fibrolipoma. Finally, the lesions were poor of fibrous tissues and immature mesenchymal tissues, which are a typical feature of fibrous hamartoma in infants. Therefore, the histological examination of all the lesions was suggestive of congenital perineal lipoma.

According to PubMed research using the MeSH terms as previously described, to date, a few more than 50 cases of congenital perineal lipoma have been reported (9, 10). To date, only seven reports on perineal lipoma associated with accessory labioscrotal fold in female newborns have been published, for a total of fifteen female patients (9–15).

All our patients had one or more anomalies associated with congenital perineal lipoma. Among the variety of congenital perineal masses, perineal lipomatous malformations are most commonly associated with other congenital abnormalities: mostly genitourinary or anorectal malformations but rarely with musculoskeletal anomalies (2, 4–6, 16). In our case series, all had an external genitalia abnormality; moreover, case number 2 had a peculiarity that the perineal lipoma was associated with skeletal malformation in addition to the accessory labioscrotal fold, which had been only reported once (17). Perineal lipomas occur infrequently in association with scrotal anomalies and are seldom reported in neonates (18). Developmental scrotal anomalies are uncommon, with accessory scrotum being the least frequent: to date less than sixty cases have been reported and its main feature is the presence of additional scrotal tissue under a normally developed scrotum, without any testis within (2, 3, 19). This anomaly occurs as an isolated abnormality in a quarter of the cases or, more commonly, concomitant with other anomalies, in few cases genitourinary or anorectal malformations (7, 19), and in about 80% of cases it is associated to contiguous subcutaneous tumors, mostly lipoma (2, 16, 19). Therefore, like in one-to-one correspondence, perineal benign tumor in nearly all cases is associated with accessory scrotum or accessory labioscrotal fold, and when concomitant, the two lesions appear contiguous with wrinkled skin, which covers a portion or the whole mass (3, 11, 12, 16). The frequent coexistence of these two lesions in a close anatomic relationship suggests the possibility of lipomatous mass involvement in the pathogenesis of accessory scrotum/labioscrotal fold (or vice versa).

The scrotum and labia majora have a common embryologic origin: from the two genital swellings that appear on both sides of the genital tubercle on the 4th week of gestation and migrate to the caudal portion after 12 weeks of gestation. In a male fetus, the labioscrotal swellings fuse and become the scrotum, and the midline forms the scrotal raphe (2, 3, 6, 11, 19). Scrotal ectopia is believed to be caused by abnormal migration of the ipsilateral labioscrotal swelling (16), although the etiology of accessory scrotum/labioscrotal fold remains unclear. First attempts to elucidate this process date to some decades ago: Lamm and Kaplan suggested that early division of labioscrotal swelling, with consecutive abnormal migration of the inferior portion to the midline, resulted in accessory scrotum (16); according to Takayasu, accessory scrotum develops from an early division and teratoid growth of pluripotential labioscrotal elements (3). Subsequently, Sule et al. in (16) presented an exhaustive literature review of accessory labioscrotal fold and its frequent association with perineal lipoma (2), and the authors theorized that accessory scrotum usually develops as a consequence of an interposed mesenchymal tissue (e.g., the developing subcutaneous tumor), which disrupts the continuity of the developing caudal labioscrotal swelling (16). This hypothesis, however, does not satisfactorily explain the etiology of accessory scrotum/labioscrotal fold, because the anomaly may occur without any concomitant perineal tumor. Furthermore, perineal lipoma may arise related to other scrotal anomalies such as ectopic scrotum (2) or is associated with further urogenital abnormalities such as hypospadias (4), cryptorchidism (5), and cervovaginal duplication (6), or it may arise as an isolated lesion (1). More recently, in 2014, Iida et al., similar to Sule, hypothesized that an aberrant adipose tissue in the perineum during the early phase of gestation disturbed the fusion of the labioscrotal folds, and, in turn, the remaining tissue became the accessory scrotum (19).

Also, the etiopathogenesis of perineal masses remains unknown. During embryogenesis, the perineal area is formed by the tip of the urorectal septum, which divides the cloaca into a ventral part (the urogenital sinus) and a dorsal part (the rectum and proximal anal canal); this process may explain the anorectal or urogenital anomalies associated with perineal lipoma, which may be caused by the abnormal development of urorectal septum as previously postulated (6). More than 80% of perineal lipomas occur with other anomalies (20). As highlighted, lipomatous masses might be associated with accessory scrotum in male or labium major in female newborn patients (11–13, 16, 20). Histological differences between labioscrotal skin and non-labioscrotal skin are defined by the presence of smooth muscle cells under the scrotal dermis microscopically, whereas macroscopic differences are hyper-pigmentation and rugated skin of the labioscrotal fold (19). The three cases presented in this study all showed smooth muscle bundles in the dermal collagen.

In conclusion, following an accurate review of the literature, a perineal lipoma can be concomitant with various external genitalia anomalies, and the location of the perineal lipoma is directly related to the associated scrotal anomalies. We present three cases of accessory scrotum/labioscrotal fold, all concurrent with perineal lipoma. In addition, the patient number 1 presents a penoscrotal transposition, a feature that supports the hypothesis of an obstacle to labioscrotal folds fusion. Therefore, given our three cases and the high frequency of lipoma concomitant to accessory scrotum/labioscrotal fold, well documented in the literature, we agree with Sule and Iida, and we speculate that when perineal lipoma and accessory scrotum/labioscrotal fold are concomitant, the development of lipoma might be closely related to abnormal development of labioscrotal swelling and is likely to be related to the pathogenesis of an accessory scrotum. The abnormal adipose tissue in the perineum during the first weeks of gestation disturbs the continuity of the developing caudal labioscrotal swelling and the fusion of the labioscrotal folds (in male patients), while the remaining tissue would become the accessory scrotum. Further research should be conducted to unravel the embryological relationship between perineal lipoma and abnormal scrotal development and the etiology of isolated labioscrotal fold anomalies and perineal lipoma.

## Data Availability Statement

The raw data supporting the conclusions of this article will be made available by the authors, without undue reservation.

## Ethics Statement

Ethical review and approval was not required for the study on human participants in accordance with the local legislation and institutional requirements. Written informed consent to participate in this study was provided by the participants’ legal guardian/next of kin.

## Author Contributions

FT: data acquisition and drafting of the manuscript. CC and AB: histopathological analyses. FF: surgical contribution. MG and FM: critical revision. All authors contributed to the article and approved the submitted version.

## Conflict of Interest

The authors declare that the research was conducted in the absence of any commercial or financial relationships that could be construed as a potential conflict of interest.

## Publisher’s Note

All claims expressed in this article are solely those of the authors and do not necessarily represent those of their affiliated organizations, or those of the publisher, the editors and the reviewers. Any product that may be evaluated in this article, or claim that may be made by its manufacturer, is not guaranteed or endorsed by the publisher.
